# Cooperative contributions of the *klf1* and *klf17* genes in zebrafish primitive erythropoiesis

**DOI:** 10.1038/s41598-023-39196-1

**Published:** 2023-08-10

**Authors:** Hiroaki Suzuki, Tomotaka Ogawa, Shigeyoshi Fujita, Ryota Sone, Atsuo Kawahara

**Affiliations:** Laboratory for Developmental Biology, Center for Medical Education and Sciences, Graduate School of Medical Science, University of Yamanashi, 1110 Shimokato, Chuo, Yamanashi 409-3898 Japan

**Keywords:** Erythropoiesis, Differentiation

## Abstract

Krüppel-like transcription factors (Klfs), which are characterized by the three conserved C-terminal zinc fingers, are involved in various biological processes, such as haematopoiesis and angiogenesis. However, how the Klf family of transcription factors cooperate in organogenesis remains elusive. During zebrafish embryogenesis, both *klf1* and *klf17* are expressed in the intermediate cell mass (ICM), where primitive erythroid cells are produced. Using CRISPR–Cas9 genome editing technology, we established *klf1*-*klf17* double mutant zebrafish to investigate the functionally interactive roles of the *klf1* and *klf17* genes. The *klf1*-*klf17* mutant exhibited a diminished number of circulating primitive erythroid cells at 2 days postfertilization (dpf), while *klf1* or *klf17* single mutants and wild-type embryos produced comparable numbers of primitive erythroid cells. Circulating erythroid cells from the *klf1*-*klf17* mutant possessed larger nuclei at 2 dpf than wild-type cells, suggesting the impairment of primitive erythroid cell maturation. The expression of the erythroid cell maturation markers *band3* and *mitoferrin*, but not the haematopoietic progenitor markers *c-myb* and *scl*, was decreased in the *klf1*-*klf17* mutant at 1 dpf. Thus, these results illustrate the cooperative function of *klf1* and *klf17* in the maturation processes of zebrafish primitive erythroid cells.

## Introduction

The molecular mechanism underlying haematopoietic development is well conserved between zebrafish and mammals^1^. In fact, haematopoietic cells are produced from two consecutive waves of primitive and definitive haematopoiesis during vertebrate embryogenesis^2^. Although myeloid precursors originate from the anterior lateral mesoderm (ALM) during zebrafish primitive haematopoiesis, erythroid precursors originate from the posterior lateral mesoderm (PLM)^1,3^, which later becomes the blood island/intermediate cell mass (ICM). Erythroid precursors differentiate into primitive erythroid cells in the ICM and migrate into vascular vessels to circulate at approximately 25 h postfertilization (hpf), when the heartbeat begins. Haematopoietic stem cells (HSCs) in definitive haematopoiesis are derived from the aorta-gonad-mesonephros (AGM) and start to produce various differentiated blood cells, including definitive erythroid, myeloid and lymphoid cells, at approximately 3–5 days postfertilization (dpf) in the caudal haematopoietic tissue (CHT) and later in the kidney^4^.

Krüppel-like transcription factors (Klfs), which possess Cys_2_/His_2_ zinc fingers at the C-terminus that interact with a CACCC box in the promoter region of their target genes, function as key transcriptional regulators in various organogenesis processes, including haematopoiesis^5^. The *Klf1* gene known as *EKLF* in mice is specifically expressed in the yolk sac blood island and foetal liver; both sites generate primitive erythroid cells^6,7^. *Klf1*-deficient mice die from β-thalassemia at embryonic Day 14 (E14.5); however, the developmental function of the *klf1* gene in other vertebrates is not fully understood. During mouse embryogenesis, *Klf6* is widely expressed in several tissues, including the kidney, heart and lung. *Klf6*-deficient mice die at E12.5 from defects in erythropoiesis and yolk sac vascularization^8^. Thus, multiple *Klf* genes contribute to the control of mammalian haematopoietic development.

We have previously isolated zebrafish *klf17*/*biklf* (*blood island-enriched Krüppel-like factor*), which is expressed in the blood island/ICM^9^. Although transient knockdown analysis of the *klf17* gene using antisense morpholino injection caused defects in primitive erythropoiesis^10^, zebrafish with CRISPR–Cas9-mediated *klf17* deficiency exhibited blood circulation without obvious haematopoietic defects^11^. In contrast, both hatching defects and abnormal lateral neuromast depositions are observed in the mutant^11^. Other *klf* genes, such as *klf1*, *klf2a*, *klf3*, *klf6a* and *klf8*, are expressed in the ICM^12^, raising the possibility that these genes serve redundant functions in zebrafish haematopoiesis. A recent study has shown that knockdown of *klf3* or *klf6a* using antisense morpholinos causes fewer mature erythroid cells^12^. However, genetic ablations of ICM-expressed *klf* genes, except for *klf17*, have not been analysed during zebrafish haematopoietic development.

At present, the function of ICM-expressed *klf* genes in haematopoietic development in zebrafish is not clear. Therefore, we established the *klf1* single mutant and *klf1-klf17* double mutant using CRISPR–Cas9 and investigated the morphological phenotypes of the mutants during zebrafish haematopoiesis. The *klf1* mutant and *klf17* mutant exhibited normal blood circulation at 2 dpf, whereas the number of circulating blood cells was reduced in the *klf1-klf17* double mutant. Primitive erythroid cells from the *klf1-klf17* mutant contained a large nucleus at 2 dpf, suggesting maturation defects in primitive erythroid cells. These results suggest that the function of the *klf1* and *klf17* genes is required for zebrafish primitive erythropoiesis.

## Results

### Establishment of the *klf1* mutant and *klf1*-*klf17* double mutant and phenotypic analysis of these mutants in zebrafish haematopoietic development

Both the *klf1* and *klf17* genes are specifically expressed in the blood island/ICM during zebrafish embryogenesis^9,13^. The expression of *klf1* was maintained in the CHT, whereas the expression of *klf17* was diminished in the CHT. The production of primitive erythroid cells is observed in *klf17*-deficient fish as well as wild-type fish^11^. Because there has been no report on the phenotype of *klf1*-deficient zebrafish in haematopoietic development, we established *klf1* single mutants and *klf1*-*klf17* double mutants using CRISPR–Cas9 genome editing technology. The *klf1* mutant allele *uy19* possesses a deletion of 35 base pairs (bp). The Klf1 mutant protein was thus functionally disrupted because it lacked most of the C-terminal domain, including the three zinc fingers (Fig. S1 and S2).

**Figure 1:**
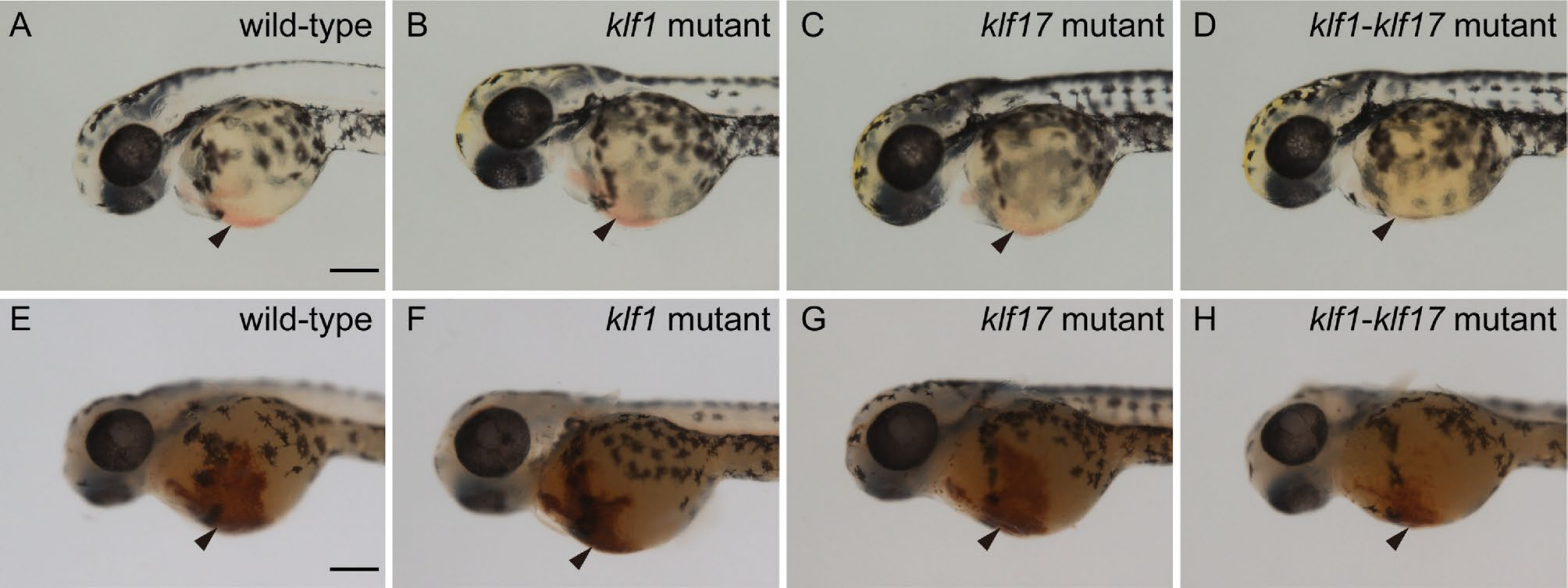
The *klf1-klf17* double mutant exhibited impairment of primitive erythropoiesis. (**A**–**D**) Live embryos at 48 hpf. The red colour of blood-circulating erythroid cells (arrowheads) was faint in the *klf1*-*klf17* mutant (*klf1*^-/-^*klf17*^-/-^) (**D**) compared to those in the wild-type (*klf1*^+/-^*klf1*7^+/+^) (**A**), *klf1* mutant (*klf1*^-/-^*klf1*7^+/-^) (**B**) and *klf17* mutant (*klf1*^+/+^*klf1*7^-/-^) (**C**). (**E**–**H**) Detection of haemoglobin (arrowheads) at 48 hpf. Haemoglobin production was examined by *o*-dianisidine staining. Haemoglobin production in the yolk was reduced in the *klf1*-*klf17* mutant (**H**) compared to the wild-type (**E**), *klf1* mutant (**F**) and *klf17* mutant (**G**). Genotyping was performed by genomic PCR using locus-specific primers. Scale bar, 200 μm (**A**, **E**).

**Figure 2:**
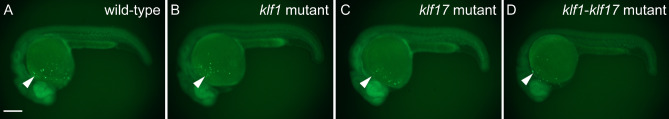
Primitive myelopoiesis in the *klf1*-*klf17* mutant. (**A**) Wild-type (*klf1*^+/-^*klf17*^+/+^), 22 hpf. (**B**) *klf1* (*klf1*^-/-^*klf17*^+/+^) mutant, 22 hpf. (**C**) *klf17* (*klf1*^+/-^*klf17*^-/-^) mutant, 22 hpf. (**D**) *klf1*-*klf17* (*klf1*^-/-^*klf17*^-/-^) mutant, 22 hpf. Primitive myeloid cells were visualized as *lyz*:EGFP-positive cells on the yolk and were comparable in the wild-type, *klf1* mutant, *klf17* mutant and *klf1*-*klf17* mutant (arrowheads). Genotyping of individual embryos was performed by genomic PCR. Scale bar, 200 μm (**A**).

We first examined the production of primitive haematopoietic cells in the *klf1* single mutant, *klf17* single mutant and *klf1*-*klf17* double mutant at 48 hpf. Primitive haematopoiesis in vertebrates mainly produces primitive erythroid and myeloid cells, and most blood-circulating haematopoietic cells in zebrafish are primitive erythroid cells. The presence of primitive erythroid cells around the heart at 48 hpf was observed by the presence of a red colour in the *klf1* mutant (n = 3) as well as wild-type (n = 6) or the *klf17* mutant (n = 3) (Fig. 1), whereas the red colour of primitive erythroid cells in the *klf1*-*klf17* mutant (n = 5) was faint. Consistently, the number of blood-circulating erythroid cells in the trunk of the *klf1*-*klf17* mutant was reduced compared to those of the wild-type, *klf1* mutant and *klf17* mutant (Supplemental Videos S1–S4). We counted the number of blood-circulating erythroid cells in the intersegmental vessels (ISVs) (Supplemental Videos S5, S6). We found that the number of blood-circulating erythroid cells in the ISV of *klf1*-*klf17* mutant (n = 5) was reduced compared to that of wild-type (n = 6) (Supplemental Fig. S3). Next, we examined haemoglobin production in primitive erythroid cells by *o*-dianisidine staining. Haemoglobin production at 48 hpf was decreased in the *klf1*-*klf17* mutant, while haemoglobin production was comparable among the wild-type, *klf1* mutant and *klf17* mutant. It has been shown that both *mitoferrin*-deficient zebrafish and *alas2*-deficient zebrafish exhibit anaemia, and the haemoglobin production defect in *mitoferrin*-deficient zebrafish, but not *alas2*-deficient zebrafish, is restored by the treatment with hinokitiol (1 μM) and ferric citrate (10 μM)^14^. We confirmed that haemoglobin production was decreased in the *klf1-klf17* mutant (Supplemental Fig. S4E, wild-type vs *klf1-klf17* mutant). Haemoglobin production of the *klf1-klf17* mutant treated with dimethyl sulfoxide (DMSO) (n = 10) or the *klf1-klf17* mutant treated with hinokitiol (1 μM) and ferric citrate (10 μM) (n = 8) equivalently decreased compared to that of the wild-type treated with DMSO (n = 12) or the wild-type treated with hinokitiol (1 μM) and ferric citrate (10 μM) (n = 11) (Supplemental Fig. S4). The status of primitive myelopoiesis was evaluated by the expression of the *lyz:EGFP* transgene^15^. The number of *lyz:EGFP*-positive cells was comparable among the wild-type (n = 7), *klf1* mutant (n = 7), *klf17* mutant (n = 5) and *klf1*-*klf17* mutant (n = 4) (Fig. 2). Consistently, the number of *lyz*-positive cells visualized by whole-mount in situ hybridization (WISH) was comparable between the wild-type (n = 7) and *klf1*-*klf17* mutant (n = 3) (Supplemental Fig. S5. Because the *klf1*-*klf17* mutant, but not the *klf1* mutant or *klf17* mutant showed primitive erythropoiesis defects, we focused on the cooperative function of *klf1* and *klf17* in primitive erythropoiesis.

### Blood circulation and maturation of primitive erythroid cells in the *klf1*-*klf17* mutant

**Figure 3:**
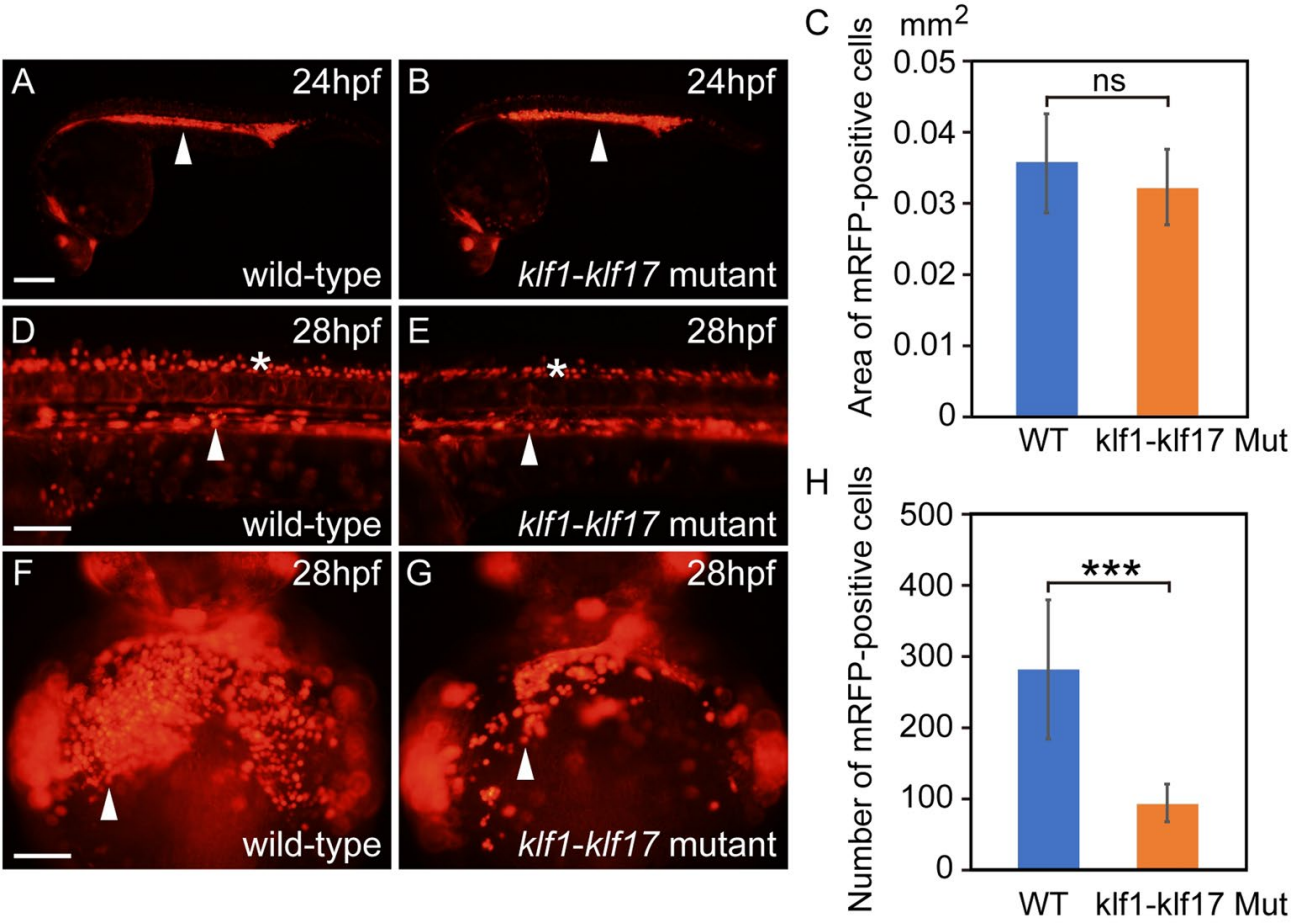
Blood circulation in wild-type and *klf1-klf17* mutant embryos. Fluorescence microscopy images of the wild-type (**A**, **D**, **F**) and *klf1*-*klf17* mutant (**B**, **E**,** G**). Lateral views, with the anterior to the right (**A**, **B**, **D**, **E**). Ventral views, with the anterior at the top (**F**, **G**). (**A**, **B**) *gata1*:mRFP-positive erythroid cells (white arrowheads) in the ICM at 24 hpf. Genotyping of individual embryos was performed by genomic PCR. Scale bar, 200 μm (**A**). (**C**) Area existing *gata1*:mRFP-positive erythroid cells in the ICM at 24 hpf. The area of *gata1*:mRFP-positive erythroid cells in the ICM was comparable between wild-type (n = 3) and *klf1*-*klf17* mutant (n = 7). The data shown are the mean ± standard deviation (SD). ns, not significant. (**D**, **E**) *gata1*:mRFP-positive erythroid cells (white arrowheads) in the dorsal aorta and *gata1*:mRFP-positive neurons (asterisks) in the neural tube at 28 hpf. Scale bar, 100 μm (**D**). (**F**, **G**) *gata1*:mRFP-positive erythroid cells (white arrowheads) on the yolk at 28 hpf. Scale bar, 100 μm (**F**). (**H**) Number of *gata1*:mRFP-positive erythroid cells on the yolk. The number of *gata1*:mRFP-positive erythroid cells was reduced in the *klf1*-*klf17* mutant (n = 7) compared to that of wild-type (n = 9). The data shown are the mean ± standard deviation (SD). *** P < 0.001 was considered significant.

Because erythroid cells can be visualized by the erythroid cell-specific expression of *gata1*:mRFP^15^, we used this strategy to examine the blood circulation of primitive erythroid cells in wild-type and *klf1*-*klf17* mutant zebrafish. The area of *gata1*:mRFP-positive erythroid cells in the ICM was comparable between the wild-type (n = 3) and *klf1*-*klf17* mutant (n = 7) at 24 hpf (Fig. 3A,B,C). The *gata1*:mRFP-positive erythroid cells in wild-type (n = 3) and *klf1*-*klf17* mutant (n = 7) migrated into the dorsal aorta and started circulation on the yolk at 28 hpf (Fig. 3D,E, Supplemental Videos S7, S8). The number of *gata1*:mRFP-positive neurons in the neural tube was comparable between wild-type and *klf1*-*klf17* mutant. On the other hand, the number of *gata1*:mRFP-positive erythroid cells on the yolk of the *klf1*-*klf17* mutant (n = 7) was reduced compared to that of wild-type (n = 9) (Fig. 3F,G,H).

**Figure 4:**
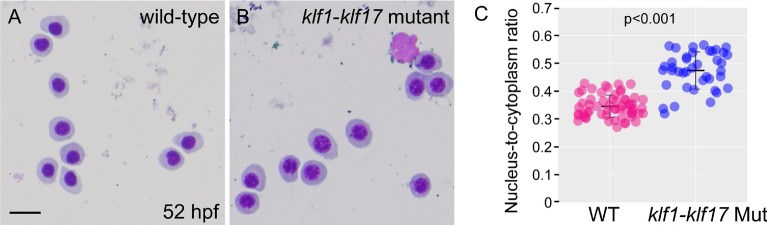
Wright–Giemsa staining of erythroid cells from wild-type and *klf1-klf17* mutant embryos. (**A**, **B**) Wright–Giemsa staining at 52 hpf. Erythroid cells in the *klf1*-*klf17* mutant (**B**) had larger nuclei and more basophilic cytoplasm than wild-type cells (**A**) at 52 hpf. Scale bar, 10 μm (**A**). Genotyping was performed by genomic PCR using locus-specific primers. (**C**) Scatter plots of the nucleus-to-cytoplasm (N:C) ratio in the wild-type (n = 6) and the *klf1*-*klf17* mutant (n = 4) at 52 hpf. The N:C ratio in the *klf1*-*klf17* mutant was larger than that in the wild-type. The central horizontal lines indicate the mean values. The data shown are the mean ± standard deviation (SD). Each wild-type and *klf1*-*klf17* mutant sample contained 10 cells. *P* < 0.001 was considered significant.

The morphological phenotype of erythroid cells in the *klf1*-*klf17* mutant was determined by Wright–Giemsa staining, which is useful for the characterization of the developmental stages of the erythroid lineage. The nucleus-to-cytoplasm ratio of erythroid cells is decreased during erythroid cell maturation^16^. We quantified the nucleus-to-cytoplasm (N:C) ratio in the wild-type (n = 6) and the *klf1*-*klf17* mutant (n = 4) at 52 hpf (Fig. 4). We found that the size of erythrocyte nuclei from the *klf1*-*klf17* mutant was larger than that of wild-type at 52 hpf (Fig. 4C), suggesting maturation defects of erythroid cells during zebrafish primitive erythropoiesis.

### Expression of haematopoietic genes in the *klf1*-*klf17* mutant

**Figure 5:**
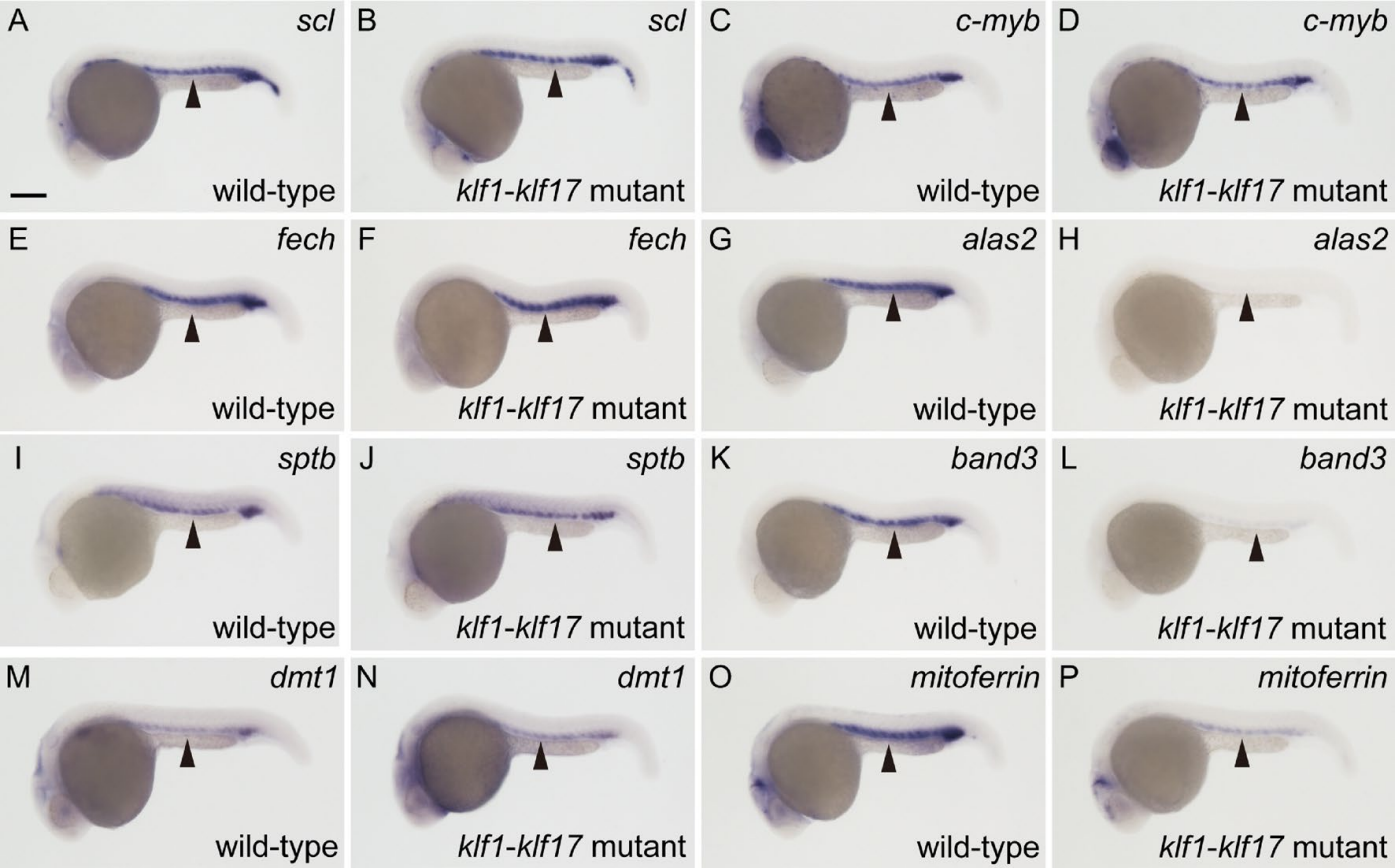
Differential expression of haematopoietic genes in the *klf1-klf17* mutant. (**A**,**C**,**E**,**G**,**I**,**K**,**M**,**O**) Wild-type embryos with wild-type alleles of *klf1* and *klf17* at 24 hpf. (**B**,**D**,**F**,**H**,**J**,**L**,**N**,**P**) *klf1*-*klf17* mutant at 24 hpf. Whole-mount in situ hybridization (WISH) with *scl* (**A**,**B**), *c-myb* (**C**,**D**), *fech* (**E**,**F**), *alas2* (**G**,**H**), *sptb* (**I**,**J**), *band3* (**K**,**L**), *dmt1* (**M**,**N**) and *mitoferrin* (**O**,**P**)*.* All pictures show lateral views, with the anterior to the left. Scale bar, 200 μm (**A**). Arrowheads indicate the position of the ICM. The expression of *scl*, *c-myb*, *fech*, *sptb* and *dmt1* at 24 hpf was comparable between the wild-type and *klf1*-*klf17* mutant. In contrast, the expression of *alas2, band3* and *mitoferrin* was decreased in the *klf1*-*klf17* mutant. Genotyping of individual embryos was performed by genomic PCR.

**Figure 6:**
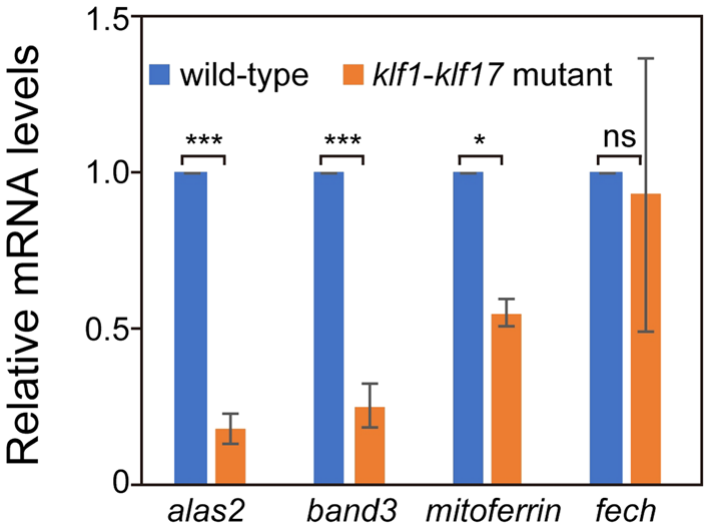
Expression levels of haematopoietic genes in the *klf1-klf17* mutant. Quantitative real-time PCR (qPCR) represents the indicated gene expression relative to that of the *tubulin α1* (*tuba1*) gene. *alas2*, *** p < 0.001. *band3*, *** p < 0.001. *mitoferrin*, * p < 0.05. *fech*, ns, not significant. The results are expressed as the mean ± standard deviation (SD). The *fech* expression level was comparable between the wild-type and the *klf1*-*klf17* mutant, whereas the expression levels of *alas2*, *band3* and *mitoferrin* were diminished in the *klf1*-*klf17* mutant.

To examine what kind of genes are affected in the *klf1*-*klf17* mutant, we performed WISH analysis for haematopoietic genes. The myeloid cell-specific genes *cebp1* (wild-type: n = 9, mutant: n = 8) and *l-plastin* (wild-type: n = 9, mutant: n = 11) were expressed at comparable levels in the wild-type and *klf1*-*klf17* mutant at 20 hpf (Fig. S6). The expression of the haematopoietic progenitor genes *c-myb* (wild-type: n = 11, mutant: n = 11), *draculin* (wild-type: n = 11, mutant: n = 12) and *scl* (wild-type: n = 11, mutant: n = 12) and the erythroid cell-specific genes *βe1-globin* (wild-type: n = 7, mutant: n = 7) and *gata1* (wild-type: n = 7, mutant: n = 9) at 24 hpf was comparable between wild-type and *klf1*-*klf17* mutant embryos (Fig. 5 and Fig. S6). During erythroid cell maturation, *gata1* expression is downregulated at 48 hpf^17^. We found that the expression of *gata1* in circulating erythroid cells of the *klf1*-*klf17* mutant (n = 9) was maintained at a high level compared to that in wild-type cells (n = 11) (Fig. S7). The expression of the haem synthesis enzyme genes *5*′*-aminolevulinate synthase 2* (*alas2*) (wild-type: n = 20, mutant: n = 19), *coproporphyrinogen oxidase* (*cpo*) (wild-type: n = 9, mutant: n = 10) and *porphobilinogen deaminase* (*pbgd*) (wild-type: n = 11, mutant: n = 9) was decreased in the *klf1*-*klf17* mutant, whereas the expression of *ferrochelatase* (*fech*) (wild-type: n = 7, mutant: n = 8) and *uroporphyrinogen decarboxylase* (*urod*) (wild-type: n = 7, mutant: n = 8) was not affected. The expression of erythroid anion exchanger *band3* (wild-type: n = 19, mutant: n = 21) was reduced in the *klf1*-*klf17* mutant, while the expression of the cytoskeletal protein gene β*-spectrin* (*sptb*) (wild-type: n = 11, mutant: n = 13) was not affected. The expression of the mitochondrial iron transporter *mitoferrin* (wild-type: n = 7, mutant: n = 8), but not *divalent metal transporter 1* (*dmt1*) (wild-type: n = 12, mutant: n = 12), was diminished in the *klf1*-*klf17* mutant. Quantitative real-time PCR (qPCR) analysis confirmed that the expression of the erythrocyte genes *alas2*, *band3* and *mitoferrin*, but not *fech*, was diminished in the *klf1*-*klf17* mutant (Fig. 6). Thus, the levels of the erythroid cell maturation markers *band3* and *mitoferrin*, but not the haematopoietic progenitor genes *c-myb* and *scl*, were decreased in the *klf1*-*klf17* mutant.

## Discussion

In this study, we established *klf1-klf17* double-deficient zebrafish with erythroid cell maturation defects and presented the cooperative function of *klf1* and *klf17* in zebrafish primitive erythropoiesis. Both zebrafish *klf1* and *klf17* genes are predominantly expressed in the blood island/ICM where primitive erythroid cells are produced^9,13^. In mice, *Klf1* is expressed in yolk sac blood islands, while the expression of *Klf17* during mouse embryogenesis is not well characterized. *Klf1*-deficient mice exhibit a lethal anaemic phenotype^6,7^. In contrast, *klf1*-deficient zebrafish and *klf17*-deficient zebrafish did not show obvious primitive haematopoietic defects (Fig. 1). We found that the number of primitive erythroid cells was reduced in the *klf1*-*klf17* double mutant (Fig. 1, 3 and Fig. S3), whereas primitive myeloid cells derived from ALM were produced at comparable levels in the wild-type and *klf1*-*klf17* mutant (Fig. 2 and Fig. S5). These results suggest the cooperative function of *klf1* and *klf17* in zebrafish primitive erythropoiesis. Multiple zebrafish *klf* genes, *klf1*, *klf2a*, *klf3*, *klf6a*, *klf8* and *klf17*, are expressed in the ICM^13^. Although *klf1*, *klf2a* and *klf6a* are substantially expressed in the CHT, the expression of *klf3*, *klf8* and *klf17* in the CHT is weak, suggesting that the cooperative function of *klf1*, *klf2a* and *klf6a* genes is required for definitive erythropoiesis. Because loss-of-function analyses of *klf* genes remain restricted at present, further analysis is required to investigate the relationship among the functional roles of *klf* genes in haematopoietic development.

During primitive erythroid cell maturation, haemoglobin production visualized by *o*-dianisidine staining was decreased in the *klf1*-*klf17* double mutant (Fig. 1 and Fig. S4). Consistent with such haemoglobin production defects, the expression of the haem synthesis genes *alas2*, *cpo* and *pbgd* was decreased in the *klf1*-*klf17* mutant (Fig. 5 and Fig. S6). Human ALAS2 is well known as the rate-limiting enzyme in haem biosynthesis^18^. The first 300 base pairs of the promoter sequence in the 5'-flanking region of the human *ALAS2* gene are required for maximal expression in erythroid cells and include GATA motifs and CACCC boxes^19^. Gel shift analysis and transactivation analysis revealed that human GATA1 and EKLF/KLF1 can bind to and activate the GATA motif and CACCC box, respectively, in the promoter. Because *alas2* expression was decreased in the *klf1*-*klf17* mutant, both *klf1* and *klf17* genes may be involved in the initial erythroid-specific induction of the *alas2* gene in zebrafish. The expression of *gata1* is downregulated during zebrafish primitive erythroid cell maturation^17^. Initial *gata1* induction at the ICM was comparable between the wild-type and *klf1*-*klf17* mutant, whereas *gata1* expression was maintained at a high level in the blood-circulating erythroid cells of the *klf1*-*klf17* mutant (Fig. S7). During zebrafish erythroid cell maturation, the size of the nucleus of erythroid cells is reduced^16^. The nucleus to cytoplasm ratio of erythroid cells, judged by Wright–Giemsa staining, was increased in the *klf1*-*klf17* mutant compared to the wild-type (Fig. 4). These results suggest that the maturation of primitive erythroid cells is impaired in the *klf1*-*klf17* mutant.

WISH analysis revealed that the haematopoietic progenitor genes *c-myb* and *scl* were expressed at comparable levels in the ICM of the *klf1*-*klf17* mutant. In contrast, the expression of the erythroid anion exchanger *band3* and a mitochondrial iron transporter *mitoferrin*, known erythroid cell maturation markers, was selectively decreased in the *klf1*-*klf17* mutant. As previously reported, *band3*-deficient zebrafish exhibit anaemia with dyserythropoiesis^20^, while *mitoferrin*-deficient zebrafish develop hypochromic anaemia^21^. The transcription of *Band3* mRNA, but not β*-Spectrin* mRNA, was diminished in *EKLF/KLF1*-deficient mice^22^. *Mitoferrin* expression is markedly downregulated in the *Klf1*-deficient mouse foetal liver^23^. Therefore, the impairment of the initial induction of *band3* and *mitoferrin* in primitive erythroid cells of the *klf1*-*klf17* mutant may be associated with erythroid cell circulation and/or erythroid cell maturation defects. Both *mitoferrin*-deficient zebrafish and *alas2*-deficient zebrafish exhibit anaemia^14,24^. The haemoglobin production defects in *mitoferrin*-deficient zebrafish, but not the *alas2*-deficient zebrafish, were restored by treatment with hinokitiol and ferric citrate^14^, suggesting that hinokitiol selectively affects iron transport processes in erythroid cell maturation. On the other hand, treatment with hinokitiol and ferric citrate did not restore the defects in haemoglobin production in the *klf1*-*klf17* mutant (Fig. S4). Various erythroid cell maturation defects, including the diminished expression of *alas2* and *band3*, are impaired in the *klf1*-*klf17* mutant, suggesting that hinokitiol and ferric citrate treatment is not sufficient to restore the haemoglobin production defects in the mutant. From these functional analyses of the *klf1*-*klf17* mutant, we propose that *klf1* and *klf17* cooperatively play important roles in the processes of erythroid cell maturation during zebrafish primitive erythropoiesis.

## Methods

### Ethics statement

All animal experiments were performed in accordance with institutional and national guidelines and regulations. The study was carried out in compliance with the ARRIVE guidelines^25^. The study was approved by the Institutional Animal Care and Use Committee (IACUC) of the University of Yamanashi and the Use Committee of the University of Yamanashi (Approval Identification Number: A30-25).

### Synthetic crRNA and tracrRNA, recombinant Cas9 protein, and microinjection

To disrupt the *klf1* genomic locus, we used the ready-to-use CRISPR–Cas9 system composed of CRISPR RNA (crRNA), *trans-activating* crRNA (tracrRNA) and recombinant Cas9 protein^26^. Synthetic crRNAs and tracrRNA (Supplementary Table S1) and recombinant Cas9 protein were obtained from Integrated Device Technology, Inc. (IDT). Synthetic *klf17*-crRNA1 (25 pg), *klf17*-crRNA2 (25 pg) and tracrRNA (100 pg) were coinjected together with recombinant Cas9 protein (1 ng) into 1-cell stage zebrafish embryos.

### Genotyping for the *klf1* and *klf17* loci and genomic sequencing

The *klf17*^*uy21*^ allele bears a deletion of bp 278–332 in the *klf17* coding region, and an unrelated 35 bp was inserted into the site^11^. The *klf1*^*uy19*^ allele bears a deletion of bp 220–254 in the *klf1* coding region. To prepare the genomic DNA, the embryos at the indicated stages were incubated in 108 μl of 50 mM NaOH at 98 °C for 10 min. Subsequently, 12 μl of 1 M Tris–HCl (pH 8.0) was added to the solution^26^. Genomic fragments at the targeted sites were amplified by PCR with PrimeTaq (Primetech) with the locus-specific primers listed in Supplementary Table S2. The PCR conditions were as follows: 95 °C for 3 min, and 98 °C for 10 s, 55 °C for 30 s and 72 °C for 30 s (40 cycles). To perform a heteroduplex mobility assay (HMA), the resultant PCR amplicons were electrophoresed on a 12.5% polyacrylamide gel^27^. To confirm the presence of the mutations in the *klf1* genomic locus, genomic fragments of the targeted genomic locus were amplified from the solution (1 μl) using PCR (Supplementary Table S2). The resultant PCR fragments were subcloned into the pGEM-T Easy vector (Promega), and the genomic sequences were determined by sequence analysis.

### Imaging

We counted the number of blood-circulating erythroid cells in the ISV using a high-speed camera (HAS-U1, DITECT Co. Ltd.). We established *klf1*^*uy19*^, *klf17*^*uy21*^ and *klf1 *^*uy19*^-*klf17 *^*uy21*^ mutant lines containing the transgene from Tg(*gata1*:mRFP)^*ko05*^ or containing the transgene from Tg(*lyz*:EGFP)^*ko02*^^15^. Zebrafish larvae were anaesthetized with tricaine (MS-222) and mounted in 3% methylcellulose. Zebrafish were imaged by a ZEISS Axio Zoom V16 fluorescence stereomicroscope.

### Wright–Giemsa staining and *o*-dianisidine staining

Zebrafish erythroid cells were prepared from the heart or vessels at 52 hpf and attached to slide glass as previously described^28^. Wright–Giemsa staining was performed to label erythroid cells.

Dechorionated zebrafish embryos at 48 hpf were incubated for 15 min with staining buffer (0.6 mg/ml *o*-dianisidine, 10 mM sodium acetate [pH 5.2], 0.65% hydrogen peroxide and 40% ethanol) to detect haemoglobin^10^.

### Whole-mount in situ hybridization (WISH)

We examined the expression of *scl*, *gata1*, *c-myb*, *sptb*, *fech*, *cebp1*, *l-plastin*, *dmt1*, *band3*, *alas2*, *mitoferrin*, *draculin*, *lmo2*, β*e1-globin*, *urod*, *pbgd* and *cpo*. WISH was performed as previously described^29^. Zebrafish embryos hybridized with the digoxygenin (DIG)-labelled RNA probe were incubated with alkaline phosphatase-conjugated anti-DIG antibody. To visualize the RNA probe recognized by the anti-DIG antibody, the samples were subsequently incubated with BM Purple (Roche) as the substrate. After three washes with PBST, the samples were fixed in 4% paraformaldehyde.

### RNA extraction and quantitative real-time PCR

Total RNA was isolated from wild-type and *klf1-klf17* mutant embryos at 24 hpf using TRIzol reagent (Thermo Fisher Scientific) and cDNA was synthesized using ReverTra Ace (Toyobo). Quantitative real-time PCR (qPCR) was performed with KAPA SYBR FAST Master Mix (Kapa Biosystems). Reaction conditions were as follows: 95 °C for 3 min, and 95 °C for 3 s and 62 °C for 30 s (40 cycles), and then a melting curve analysis at 95 °C for 15 s, 60 °C for 1 min, and 95 °C for 15 s. qPCR was performed in duplicate and in at least three independent experiments. Quantification of mRNA was normalized with *tubulin α1* (*tuba1*) as the housekeeping gene and calculated according to the 2 − ΔΔCT method.^30^. The primers used in this study are listed in Table S3.

### Drug treatment

Wild-type or *klf1-klf17* mutant embryos were dechorionated at 24 hpf and incubated in (i) 1% DMSO as a vehicle control, or (ii) hinokitiol (1 μM) and ferric citrate (10 μM) until 48 hpf^14^. Haemoglobin production was determined by *o*-dianisidine staining as described above.

## Supplementary Information

Supplementary Information 1.

Supplementary Video 1.

Supplementary Video 2.

Supplementary Video 3.

Supplementary Video 4.

Supplementary Video 5.

Supplementary Video 6.

Supplementary Video 7.

Supplementary Video 8.

## Data Availability

All data generated or analysed during this study are included in this published article and its Supplemental Information files. Additional raw data files can be available from the corresponding author upon request.
