# Supplementary material for: Cooperative contributions of the klf1 and klf17 genes in zebrafish primitive erythropoiesis
Source: Sci Rep. 2023 Aug 10;13:12279. doi: 10.1038/s41598-023-39196-1 (PMC10415360; doi:10.1038/s41598-023-39196-1)
Supplement: Supplementary file 1 — Supplementary Information 1. [file 41598_2023_39196_MOESM1_ESM.pdf]

Cooperative contributions of the *klf1* and *klf17* genes in zebrafish primitive erythropoiesis

Hiroaki Suzuki[1], Tomotaka Ogawa[1], Shigeyoshi Fujita[1], Ryota Sone[1] and Atsuo Kawahara[1*]

[1]Laboratory for Developmental Biology, Center for Medical Education and Sciences, Graduate School of Medical Science, University of Yamanashi, 1110 Shimokato, Chuo, Yamanashi, 409-3898, Japan

Keywords: *klf1*; *klf17*; primitive erythropoiesis; *band3*; *mitoferrin*

*Correspondence should be addressed to A.K. (akawahara@yamanashi.ac.jp)

Supplemental Figures

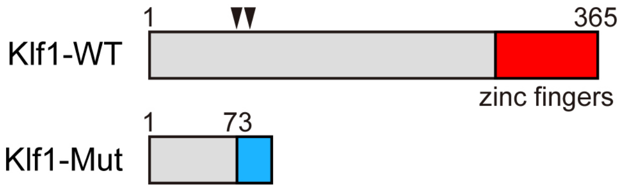

Figure S1 Molecular structures of wild-type and mutant Klf1 proteins.

The *klf1*$^{-/-}$ lesions cause premature stop codons after 34 missense amino acids (blue rectangle), starting at amino acid 74. The arrowheads indicate the CRISPR–Cas9 target sites. The zinc finger motif in Klf1 is indicated by a red rectangle.

Klf1 wild-type

MAVTQAVLPSFSNFCTNSENMKFEKGFLDLDSKDLHQSPASYKPSQFLTDEHQDDSEGCWDM

EFLLSDWASMSPERSNSQNYTPQRPTLQDQLHSPDLYQELNAPKPNRHQVCVASSLAELLPP

EATLSSSLPPDLQFNCGFLDQQAAKSYSQAENPQQFSAFPVTSNVNRDSSMGKMGKSWDFGH

YYQPAPLISFPDSKFVQTQGITMETVVFPPHHHYNFIPSYSHPRLYQQQANYVHRPPPQSHF

PSTQSMVPDSTVPPAGLESKRIRRGLVKRKATVHSCEYPGCQKTYTKSSHLKAHLRTHTGEK

PYHCTWDGCGWKFARSDELTRHFRKHTGQKPYECLLCHRAFSRSDHLALHMKRHV

Klf1 Mutant

MAVTQAVLPSFSNFCTNSENMKFEKGFLDLDSKDLHQSPASYKPSQFLTDEHQDDSEGCWDM

EFLLSDWASMSAADPTGPASQSRSLSGAERTKTKQTSSVRCKFPS

Figure S2 Amino acid sequences of wild-type and mutant proteins.

Red letters indicate the zinc finger domains. Blue letters indicate missense amino acids (34 amino acids).

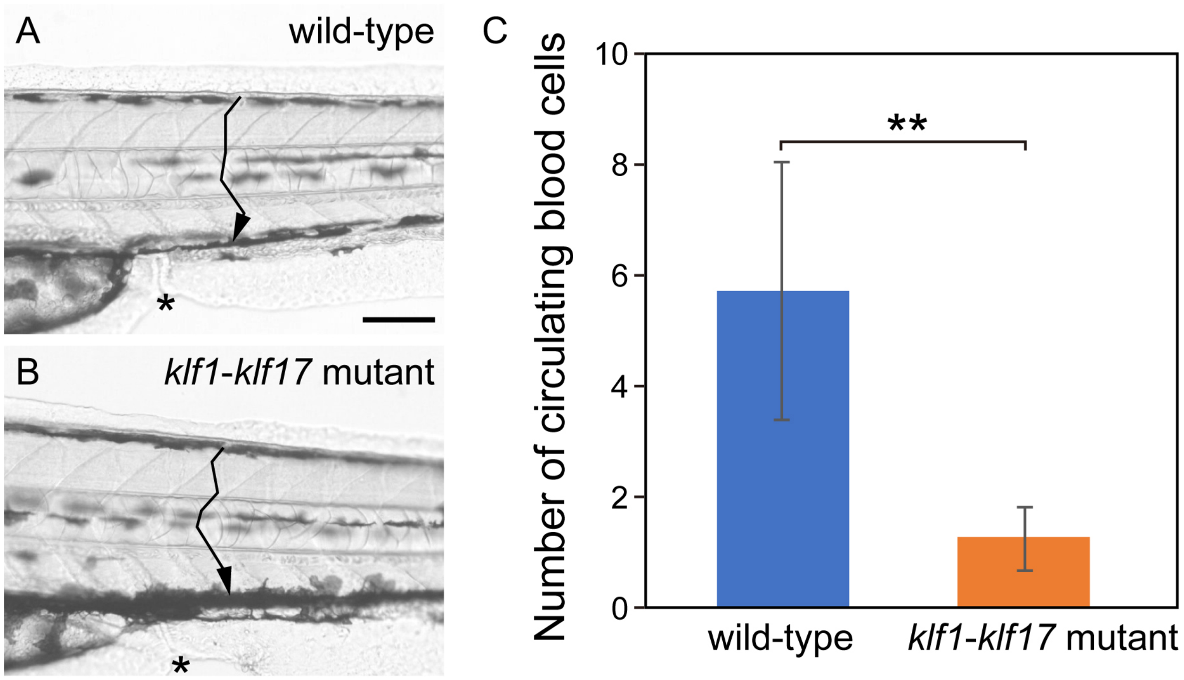

Figure S3 The number of blood-circulating erythroid cells in the ISV.

(A) Wild-type, 3 dpf. Scale bar, 100 µm. (B) *klf1-klf17* mutant, 3 dpf. We counted the number of blood-circulating erythroid cells in the ISV (arrows) as shown in Supplemental Videos S5 and S6. Asterisks indicate the position of the urogenital opening. (C) Circulating blood cells in the ISV of wild-type (n=6) and in the *klf1-klf17* mutant (n=5) were counted. The results are expressed as the mean ± standard deviation (SD). ** $P < 0.01$ was considered significant. Genotyping of individual embryos was performed by genomic PCR.

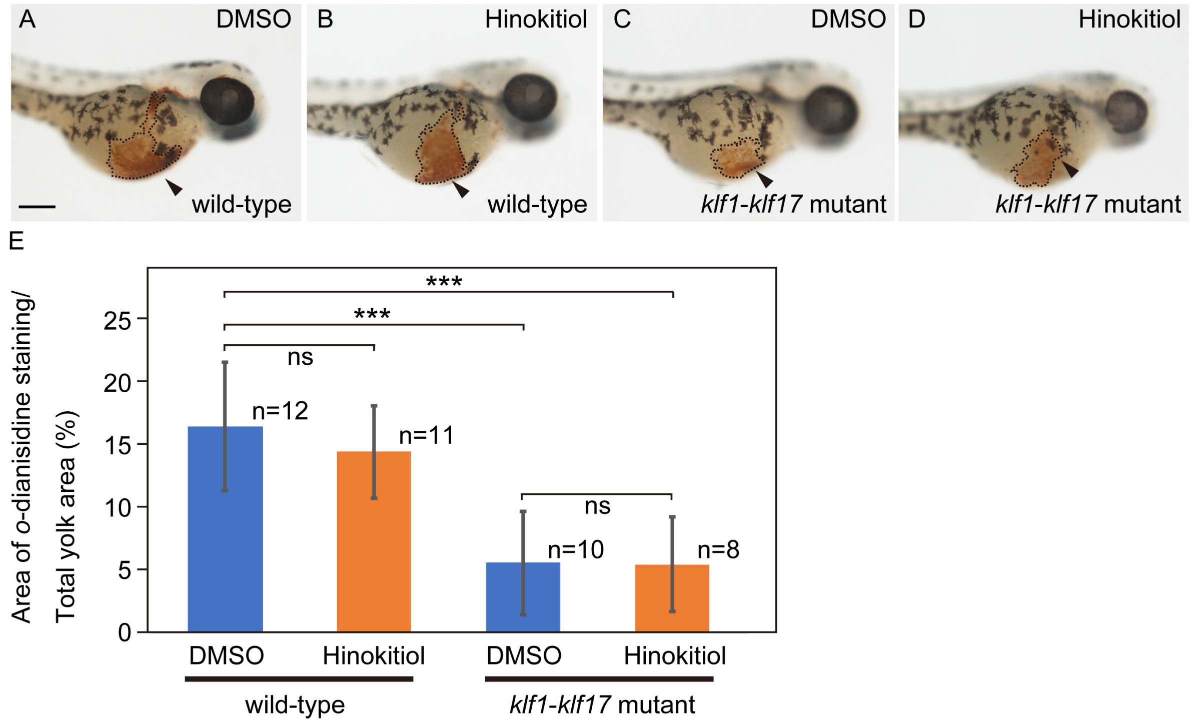

Figure S4 Haemoglobin production defects in the *klf1-klf17* mutant were not restored by treatment with hinokitiol and ferric citrate.

(A) Wild-type treated with DMSO. Scale bar, 200 μm. (B) Wild-type treated with hinokitiol (1 μM) and ferric citrate (10 μM). (C) *klf1-klf17* mutant treated with DMSO. (D) *klf1-klf17* mutant treated with hinokitiol (1 μM) and ferric citrate (10 μM). Haemoglobin production (arrowheads) was determined by *o*-dianisidine staining. (E) The area of *o*-dianisidine staining (dot line) was quantified and presented as a percentage of the total yolk area. Haemoglobin production defects in the *klf1-klf17* mutant were not restored by the treatment with hinokitiol and ferric citrate. *** $P < 0.001$ was considered significant. ns, not significant. Genotyping of individual embryos was performed by genomic PCR.

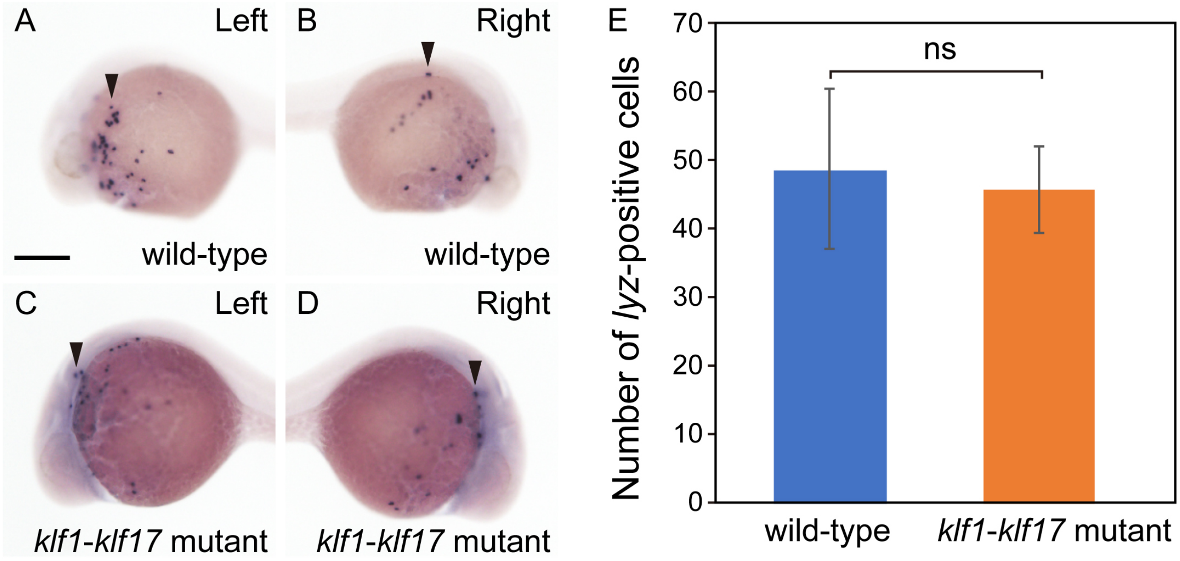

Figure S5 The number of *lyz*-positive myeloid cells in the *klf1-klf17* mutant.

(A, B) WISH analysis with *lyz* antisense RNA probe, wild-type. (C, D) WISH analysis with the *lyz* antisense RNA probe, *klf1-klf17* mutant. All pictures (right or left) show lateral views at 20 hpf. Scale bar, 200 μm (A). (E) The number of *lyz*-positive cells (as examples indicated by arrowheads) on the yolk in wild-type (n=7) and in the *klf1-klf17* mutant (n=3) were counted. The results are expressed as the mean ± standard deviation (SD). ns, not significant. Genotyping of individual embryos was performed by genomic PCR.

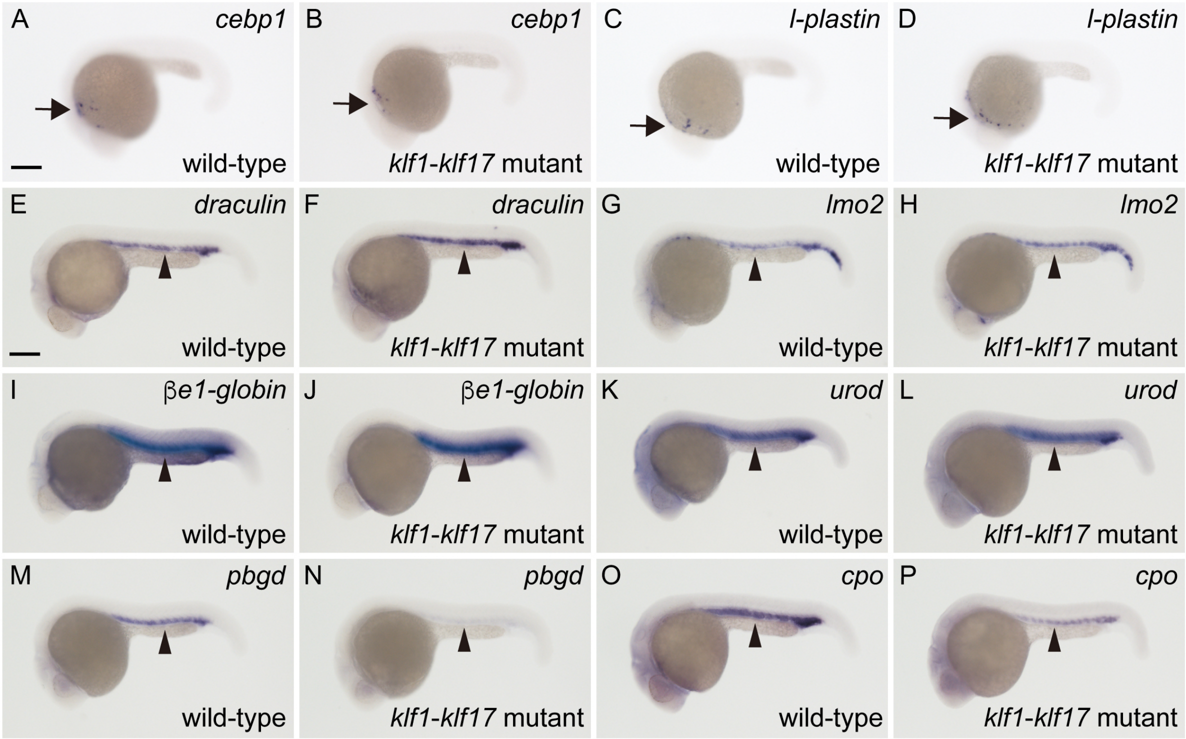

Figure S6 Differential expression of haematopoietic genes in the *klf1-klf17* mutant.

(A, C) Wild-type embryos with wild-type alleles of *klf1* and *klf17* at 20 hpf. (B, D) *klf1-klf17* (*klf1*$^{-/-}$*klf17*$^{-/-}$) mutant at 20 hpf. (E, G, I, K, M, O) Wild-type embryos containing wild-type alleles of *klf1* and *klf17* at 24 hpf. (F, H, J, L, N, P) *klf1-klf17* (*klf1*$^{-/-}$*klf17*$^{-/-}$) mutant embryos at 24 hpf. WISH was performed for *cebp1* (A, B), *l-plastin* (C, D), *draculin* (E, F), *lmo2* (G, H), *βe1-globin* (I, J), *urod* (K, L), *pbgd* (M, N) and *cpo* (O, P). All pictures show lateral views, with the anterior to the left. Scale bar, 200 μm (A, E). Arrows indicate the position of developing myeloid cells. Arrowheads indicate the position of the ICM. The expression of *cebp1* and *l-plastin* at 20 hpf was comparable between the wild-type and *klf1-klf17* mutant. The expression of *draculin*, *lmo2*, *βe1-globin*, and *urod* at 24 hpf was comparable between the wild-type and *klf1-klf17* mutant. In contrast, the expression of *pbgd* and *cpo* was decreased in the *klf1-klf17* mutant. Genotyping of individual embryos was performed by genomic PCR.

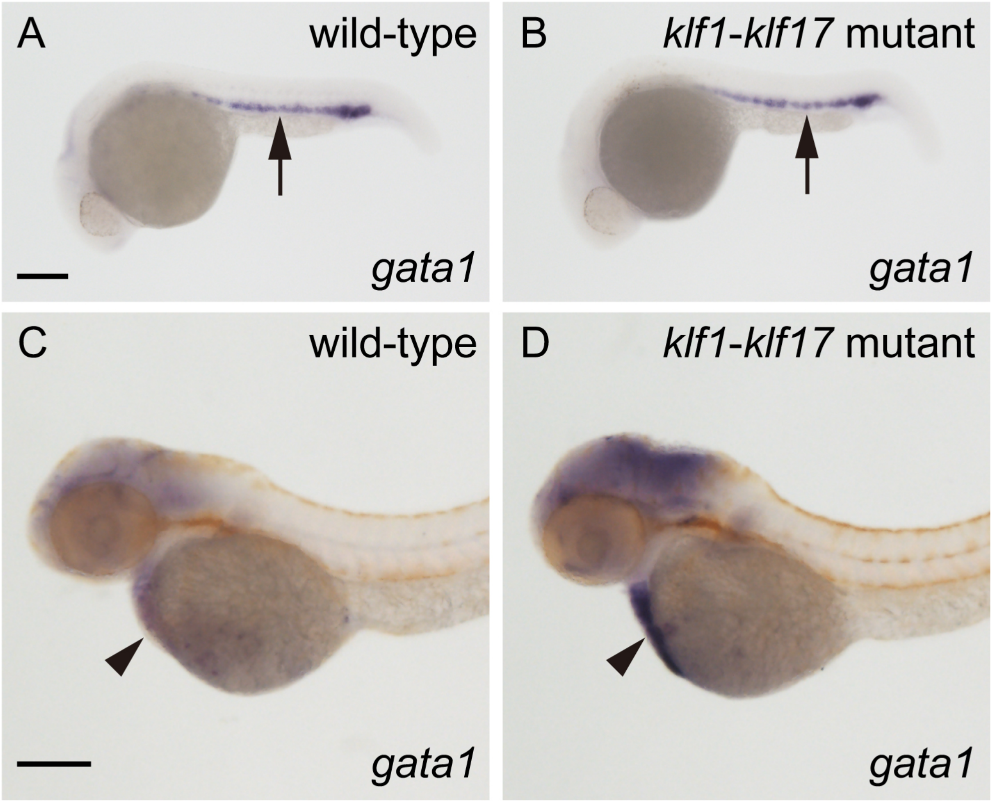

Figure S7 The expression of *gata1* in the *klf1-klf17* mutant.

(A) Wild-type at 24 hpf. (B) *klf1-klf17* mutant at 24 hpf. (C) Wild-type at 48 hpf. (D) *klf1-klf17* mutant at 48 hpf. All pictures show lateral views, with the anterior to the left. Scale bar, 200 μm (A, C). Arrows indicate the position of the ICM. Arrowheads indicate the position of circulating primitive erythroid cells. Although the *gata1* expression in the ICM was comparable between the wild-type and the *klf1-klf17* mutant, the expression of *gata1* in circulating primitive erythroid cells of the *klf1-klf17* mutant was maintained at a high level compared to that of the wild-type at 48 hpf. Genotyping of individual embryos was performed by genomic PCR.

Supplemental Table

Table S1 The targeted genomic sequences.

Targeted genomic sequences for CRISPR–Cas9

Target	Sequence (5′ to 3′)
klf1-1	GGGCCAGTATGTCACCTGAA<u>CGG</u>
klf1-2	CTCAAAACTACACTCCCCAG<u>CGG</u>

PAM sequences are underlined.

Table S2 PCR primers used in this study.

Primer name	Sequence (5′ to 3′)
klf1-HMA-F1	GATGCTGGGACATGGAGTT
klf1-HMA-R1	GGACTGTGAAGCTGGTCCT
klf17-HMA-F1	TGACAACGACAACACCTCA
klf17-HMA-R1	TGCCACAGTAGGCGTTAGG

Table S3 PCR primers for quantitative PCR analysis.

Primer name	Sequence (5′ to 3′)
alas2-F1	CCTGGTGGACACTGTACGCTC
alas2-R1	CGCCTGCACGTAGATGTTATGC
band3-F1	ATCACTTCACTGAGTGGCAT
band3-R1	CGGTCATGAACATTCTCAGAG
mitoferrin-F1	ACGAGGATTACGAGAGTTTGCC
mitoferrin-R1	TCCGGCTGTAAACTCTGCATTC
fech-F1	AGCTGGACATCGAGTACTCACAG
fech-R1	GGTCAGCTGACGAGAGCATG
tuba1-F1	GACATACCGTCAGCTGTTCCA
tuba1-R1	ACTTCTTGCCGTAGTCGACAG

Supplemental Video S1: Blood circulation of wild-type embryo at 48 hpf.

Supplemental Video S2: Blood circulation of the *klf1* mutant at 48 hpf.

Supplemental Video S3: Blood circulation of the *klf17* mutant at 48 hpf.

Supplemental Video S4: Blood circulation of the *klf1-klf17* mutant at 48 hpf.

Supplemental Video S5: Blood circulation of wild-type embryo in the ISV at 3 dpf.

Supplemental Video S6: Blood circulation of the *klf1-klf17* mutant in the ISV at 3 dpf.

Supplemental Video S7: Blood circulation of wild-type embryo on the yolk at 28 hpf.

Supplemental Video S8: Blood circulation of the *klf1-klf17* mutant on the yolk at 28 hpf.
